# A long-term maternal diet transition from high-fat diet to normal fat diet during pre-pregnancy avoids adipose tissue inflammation in next generation

**DOI:** 10.1371/journal.pone.0209053

**Published:** 2018-12-18

**Authors:** Michelle Summerfield, Yi Zhou, Tianhao Zhou, Chaodong Wu, Gianfranco Alpini, Ke K. Zhang, Linglin Xie

**Affiliations:** 1 Department of Nutrition and Food Sciences, Texas A&M University, College Station, TX, United States of America; 2 Tongji Hospital, Huazhong University of Science and Technology, Wuhan, Hubei, China; 3 Department of Medical Physiology, Texas A&M University College of Medicine, Temple, TX, United States of America; 4 Research, Central Texas Veterans Health Care System, Temple, TX, United States of America; 5 Center for Epigenetics & Disease Prevention, Institute of Biosciences & Technology, College of Medicine, Texas A&M University, Houston, TX, United States of America; University of Kansas School of Medicine, UNITED STATES

## Abstract

Recent studies have suggested that maternal high-fat (HF) diet caused inflammation changes in adipose tissue; however, it remains unclear if maternal diet intervention before pregnancy rescues such effects in offspring. To address this question, female mice were continued on a normal-fat (NF group), or a HF diet (HF group) or transitioned from a HF diet to a NF diet at 1 (H1N group), 5 (H5N group) or 9 weeks (H9N group) prior to pregnancy. Among the three intervention groups, the H9N offspring displayed less and steady body weight gain, and maintained glucose tolerance, whereas the H1N and H5N offspring showed exacerbate these phenotypes. The H1N and H5N, but not the H9N offspring, displayed adipocyte hypertrophy associated with increased expression of genes involved in fat deposition. The H1N and H5N, but not the H9N adipose tissue, displayed increased macrophage infiltration with enhanced expression of inflammatory cytokine genes. In addition, overactivation of the NF-κB and the JNK signaling were observed in the H1N adipose tissue. Overall, our study showed that a long-term but not a short- or medium-term diet intervention before pregnancy released offspring adipose tissue inflammation induced by maternal HF diet, which adds details in our understanding how the maternal environment either promotes or discourages onset of disease in offspring. Clinically, this study is of great value for providing evidence in the design of clinical trials to evaluate the urgently required intervention strategies to minimize the intergenerational cycle of obesity.

## Introduction

To date, approximately 41 million children globally are affected by childhood obesity, an increase of roughly 9 million children since the year 1990 [1–5]. These rates are expected to continue to increase at an alarming rate, and diagnosis of chronic diseases which have previously been identified only in adults, such as type 2 diabetes mellitus (adult-onset) and nonalcoholic fatty liver disease, are now being reported in pediatric clinical settings [2, 6]. By current estimates, the proportion of women who are of both childbearing age and overweight or obese (BMI >25) is two-third [7]. Maternal Pre-pregnancy BMI as well as percent ideal body weight are both significantly associated with increased risk of large for gestational weight babies and adolescent obesity [8–10].

It is well established that in-utero and early life exposure to under-nutrition or over-nutrition can disrupt normal growth and development, thus changing the offspring phenotype to one that might lead to future disease [11–17]. Recent studies have put more effort on elucidating maternal over-nutrition, which reflects the dietary habits of the Western society. In humans, babies exposed to over-nutrition during gestation have increased risks of obesity, diabetes and other complications including NAFLD [18–21]. A 3% increase in likelihood of adolescent obesity has been correlated to every 1 Kg increment in excessive gestational weight gain when children were tracked to age seven [22], indicating an urgent need to develop healthy life styles to maintain a normal body weight before pregnancy, in order to reduce the risks for offspring obesity.

Obesity has been considered a disease condition with low-grade chronic inflammation [23, 24]. The central role of adipocytes and adipose tissue macrophages (ATMs) in triggering and promoting the systemic inflammation and insulin resistance has been demonstrated recently [25]. With the theory that individual adipocyte has a threshold capacity for storage of lipid, the lipotoxicity caused by “lipid spillage” results in increased recruitment of macrophages which further change their localization and inflammatory features during obesity [26]. Thus, the enlarged adipose tissue with excessive triglycerides stored and the increased ATMs are concurrently responsible to altered release of adipokines and cytokines that control local and systemic inflammatory processes and interfere with insulin signaling [25].

With the relationship between HF diet and obesity and the incidence of adipose tissue inflammation, recent studies have collected data to understand how a maternal high-fat diet influences the inflammatory status. In animal models, offspring of mothers exposed to over-nutrition have common phenotypes that include catch-up growth, increased adiposity, impaired glucose tolerance, impaired insulin sensitivity and liver dysfunction [17, 27–30]. These pathophysiological changes are associated with induced cytokines in maternal serum and placenta involving IL-1β, TNF-α and MCP-1[31, 32]. In offspring, there is enhanced level of TNF-α in adipocytes [33] and higher IL-6, TNF-α in liver [34, 35].

Although progress has been made to understand the physiological origin of inflammation with in utero exposure to over-nutrition, there are very few of studies aiming on if and how maternal diet interventions impacts on offspring adipose tissue inflammation, which may further promote or inhibit the development of offspring obesity. This research aims to lay a framework for identifying if maternal diet intervention before pregnancy may alter adipogenic and inflammatory regulation in visceral adipose tissue of offspring. Elucidation of key regulatory mechanisms and modeling of in utero programming offers further information as to how the maternal environment either promotes or discourages onset of disease in offspring.

## Materials and methods

### Experimental design

Detailed experimental design is described previously (Table 1) [36]. Four-week-old female mice of mixed background (B6/129/SvEv) were selected for study and fed either a normal fat diet (10% kcal from fat) or a high fat diet (60% kcal from fat) for 12 weeks. The breeding pairs administered a high fat diet continued this diet through gestation and lactation (the HF group) or were transitioned to a normal fat diet at either a one-week (the H1N group), five-week (the H5N group) or nine-week (the H9N group) time window prior to pregnancy followed through gestation and lactation (at least three litters for each group). From each litter, male offspring mice were given a high fat diet for 12 weeks after weaning, to promote weight gain before being sacrificed. A separate group, birthed of the breeders who adhered to the NF diet, were continuously fed the NF diet through 12 weeks, and utilized as a reference control group. The mice were sacrificed by CO_2_ inhalation for more than 2 minutes and were subjected to cervical dislocation to ensure death. Mouse experiments were completed according to a protocol reviewed and approved by the Institutional Animal Care and Use Committee of the University of North Dakota and Texas A&M University, in compliance with the USA Public Health Service Policy on Humane Care and Use of Laboratory Animals.

**Table 1:** Study design.

Maternal Diet				Offspring Diet
Diet Groups	Pre-Pregnancy		Pregnancy & Lactation	Post-Weaning
	-9 Weeks	Transition Stage	Until Weaning	+12 Weeks
REF	NF	/	NF	NF
Normal Fat (NF)	NF	/	NF	HF
High Fat (HF)	HF	/	HF	HF
H1N	HF	-1 week	NF	HF
H5N	HF	-5 week	NF	HF
H9N	HF	-9 week	NF	HF

### Diet composition

Diet was purchased from Research Diets, LLC (New Brunswick, NJ). The normal fat diet (Cat#D12450B) had an energy density of 3.771 kcal/g (10% fat energy, 70% carbohydrate energy, and 20% protein energy). The HF diet (Cat#D12492) had an energy density of 5.157 kcal/g (60% fat energy, 20% carbohydrate energy, and 20% protein energy). The fat source is composed of 92% of lard and 8% of soybean oil.

### Energy consumption and body weight

Mice remained singly housed throughout the study and were weighed periodically. Food consumption was recorded once a week for the 12-week postnatal period as previously described (42). Both logs were tracked throughout the 12-week post-weaning HFD feeding portion of the study.

### Antibodies

Antibodies against NF-kappaB, phospho-NF-kappaB, JNK and phospho-SAPK/JNK (Thr183/Tyr185) and GAPDH were purchased from Cell Signaling Technology (USA).

### Fat mass and organ to body ratio

After the 12-week timepoint concluded, mice were humanely sacrificed with CO_2_ asphyxiation followed by cervical dislocation. Fat pads were excised from the visceral region and were weighed immediately, before being snap frozen in LN_2_. Fat pad mass was normalized against individual murine body weight.

### Adipose tissue analysis

Divided adipose tissue samples were fixed in 10% formalin/PBS. Tissue processing followed standard protocol. Briefly, tissues embedded in paraffin blocks after processing completion and then cut into 5-μm-thick slices using a microtome (Leica). Tissue was mounted either on positive pre-charged slides for HE staining, or on non-charged slides (TRU-BOND380) for IHC. HE staining was completed with Harris-modified Hematoxylin and Eosin-Y solution and staining visualized with a Leica M165FC camera at 200X magnification and Leica Application Suite X. Adipocyte diameter was measured using Image J software. Two hundred cells were randomly counted in each sample. The adipocytes of five animals from each group were counted. An average diameter was recorded for each animal.

Occupation of tissue with crown like structures was calculated using the threshold tool in the Fiji Plugin of ImageJ software. Briefly, two distinct HE stained adipose tissue images per sample (n = 5-7samples/group) were converted to an 8-bit grayscale image. A threshold was applied to measure the background occupation of crown like structures and was evaluated as percent area.

F4/80 Antibody (Cell Signaling Technology, Danvers MA) was immunostained using a VECTASTAIN Elite ABC kit for rabbit IgG (Vector Laboratory, Burlingame CA). Staining of tissue was visualized with a D2500 microscope (Leica) and images captured using a Leica M165FC camera. Percent area from 5 animals per group was calculated using the Fiji plugin of ImageJ software. Briefly, H-Dab stained slides were color deconvoluted and then a threshold applied similar to the crown like structure measurement protocol, which determined percent area. Group averages were normalized against the REF group.

### Western blots

Briefly, total protein (10 μg) was extracted from adipose tissue and standardized against a standard curve using a Peirce BCA kit (ThermoFisher, Waltham, MA). Agarose gels were run under standard conditions and transferred using a TurboBlot transfer (BioRad Laboratories, Hercules CA) system onto PVDF membranes. Membranes were imaged using a Chemidoc chemiluminescence imaging system (BioRad Laboratories, Hercules CA) and ECL reagents (EMD Millipore, Burlington MA)

### Real time PCR

Total RNA was extracted from visceral adipose tissue using Trizol reagent (Invitrogen). cDNA was synthesized using Ready Script cDNA synthesis mix (Sigma Aldrich, St. Louis, MO). Primers used were listed in Table 2. Realtime PCR was performed using a POWER SYBER Green PCR master mix from Applied Biosystems on a CFX384 Touch Real-Time PCR Detection System (BioRad Laboratories, Hercules CA). The ΔCT values were used for statistical analysis for real-time-PCR experiments. The standard deviation of the fold change in gene expression for real-time-PCR data was derived by the delta method [37].

**Table 2:** Primers used for the real-time-PCR (5’ to 3”).

*Acacb-F*	GAACCGGCTTCCTGGTTG
*Acacb-R*	TCCTCCCCTATGCCGAAA-GA
*Cd36-F*	TGGAGGCATTCTCATGCC-AG
*Cd36-R*	TTGCTGCTGTTCTTTGCC-AC
*Fabp-F*	GTGGTCCGCAATGAGTTC-AC
*Fabp-R*	GCTTGACGACTGCCTTGA-CT
*Fas F*	GGAGGTGGTGATAGCCGG-TAT
*FAS F*	TATCAAGGAGGCCCATTT-TGC
*IL10 F*	GCTCTTACTGACTGGCATGAG
*IL10 R*	CGCAGCTCTAGGAGCATGTG
*IL1b F*	GCAACTGTTCCTGAACTCAACT
*IL1b R*	ATCTTTTGGGGTCCGTCAACT
*IL6 F*	TGCCTTCTTGGGACTGAT-GC
*IL6 R*	CTGTTGTTCAGACTCTCTCCCT
*PPARg-1F*	TGGTTCAAATATGCCACC-AG
*PPARg-1R*	CCAAGTGCTGGGATTAAA-GG
*SREBP-1c F*	AGCAGTCACCAGCTTCAG-TC
*SREBP-1c R*	GGTCATGTTGGAAACCAC-GC
*Tnf F*	CCCTCACACTCAGATCATCTTCT
*Tnf R*	GCTACGACGTGGGCTACAG

### Statistical analysis

Measurements for single time points were analyzed by Fishers’ least significant difference test so multiple comparisons of differences between groups REF, NF, HF, H1N, H5N and H9N were considered. Fisher’s least significant difference test was performed by firstly carrying out one-way analysis of variance for all treatment groups. For the longitudinal data such as body weight and food consumption, a linear mixed model was used for the analysis of repeated measures with each individual mouse as a random effect. For QPCR data, all groups were normalized against the REF group. Adipocyte diameter, F4/80 quantification, and CLS calculations were firstly normalized against the REF group, and then compared using a Fisher’s least significant difference using one-way analysis of variance. A P value less than 0.05 is considered significant difference, while a P value less than 0.1 is considered marginal significance. All analyses were carried out by using SAS JMP software (SAS Institute Inc., Cary, NC, USA).

## Results

### A long-term, but not the medium-term or a short-term transition from high fat diet to normal fat diet before pregnancy slowed down offspring body weight gain and blocked the glucose intolerance induced by 12-week post-weaning HF diet

**Fig 1:**
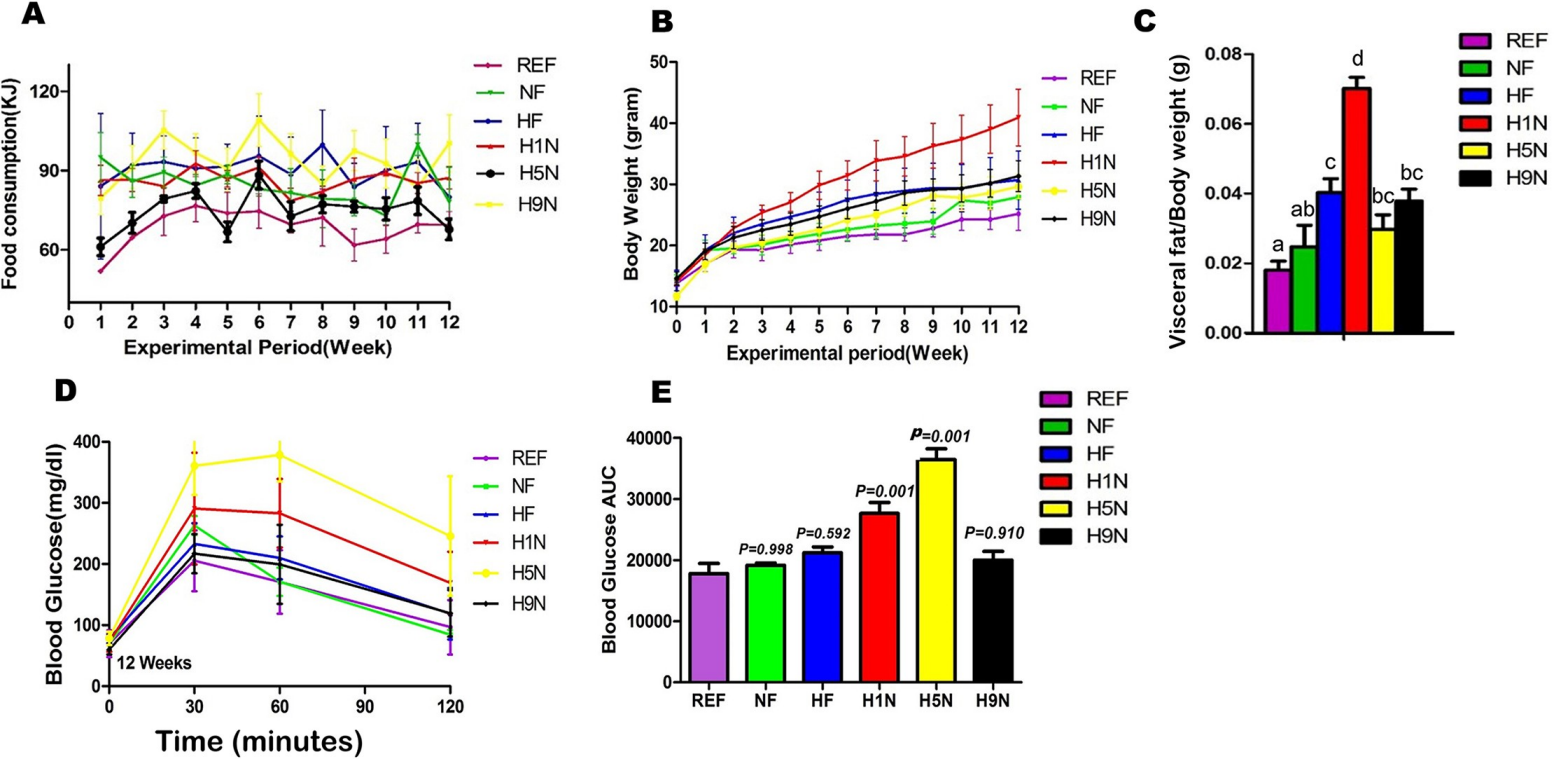
Different maternal diet intervention differentially affected the offspring body weight gain, adipose tissue mass and glucose tolerance. (A) Energy intake of the male mice from wean until week 12 showed significantly lower consumption of energy in REF group-the only post-weaning group administered NF diet. Data is reported as Mean±SEM, n = 6–10 (B) Body weight of male offspring from wean to week 12. Data is reported as Mean±SEM, n = 6–10. (C) Visceral adipose tissue mass was normalized against individual murine body weight to eliminate outliers. Data is reported as Mean±SEM, n = 6–10. Different letter indicates significance level of P<0.05 between any two groups. (D) IPGTT at week 12, H1N and H5N group had impaired glucose tolerance. Data is reported as Mean±SEM. n = 6–10 (E) H1N and H5N had significantly increased AUC (p < .01) as compared to other groups. Data is reported as Mean±SEM, n = 6–10.

To address if the different maternal diet intervention differentially affects the energy consumption of the offspring, the weekly energy intake of the offspring was recorded starting from weaning. As expected, the REF group had the lowest post-weaning energy intake, because this group is the only one exposed to post-weaning NF diet. There was no significant difference in energy intakes among the other groups on post-weaning HF diet (Fig 1A), indicating that offspring energy consumption was not dependent upon different maternal diet intervention.

At birth, there were no statistically significant differences between body weights in all groups except for the H5N group (Fig 1B). The REF group maintained the lowest body weight throughout the 12-week postnatal timepoint, correlating with continued adherence to the post-weaning NF diet. The NF group was slightly but not significantly elevated compared to the REF group by the analysis of repeated measures. Although was weaned at a significantly lower body weight, the H5N offspring seemed to experience a catch-up growth so that its body weight was similar to the NF group, but significantly higher than the REF group by the analysis of repeated measures. Both the HF and H9N consistently grew with the similar pace during the 12-week postnatal period, which were significantly elevated compared to the NF group (Fig 1B). Interestingly, the H1N group deviated in body weight beginning at week 5 and maintained a consistent and significant increase in body weight compared to all other groups (Fig 1B).

To evaluate how maternal diet effected visceral adiposity, we next measured amounts of relative visceral adipose mass when visceral adipose tissue mass was normalized against total murine body weight (Fig 1C). Clearly, the maternal HF diet significantly increased adipose tissue mass in the HF offspring comparing to the NF offspring. The H1N group had the highest level of relative adipose tissue among all treatment groups, while the relative adipose tissue of the H5N and H9N group was significantly reduced compared to the H1N group. Interestingly, both the H9N and H5N offspring retained similar amount of relative adipose tissue mass as the NF or HF offspring, however was greater than the REF offspring (Fig 1C).

Glucose tolerance was measured by performing IPGTT before termination at week 12. Fasting glucose was not significantly different between any groups. Although the glucose levels of all offspring peak between 30- to 60-minute after glucose injection, those of the H1N and H5N offspring were significantly higher than the normal level indicated in the REF group (Fig 1D). Moreover, the glucose level of the H5N group was not able to return to the basal level at 120 minutes. By evaluating the area under the curve (AUC), both the NF and HF groups were glucose tolerant at week 12 (Fig 1E). However, the H1N and H5N offspring displayed significant glucose intolerance (*p*<0.01), while the H9N offspring were able to maintain glucose tolerance.

### A long term, but not a short- or medium-term transition recovered offspring adipocyte hypertrophy induced by post-weaning HF diet

**Fig 2:**
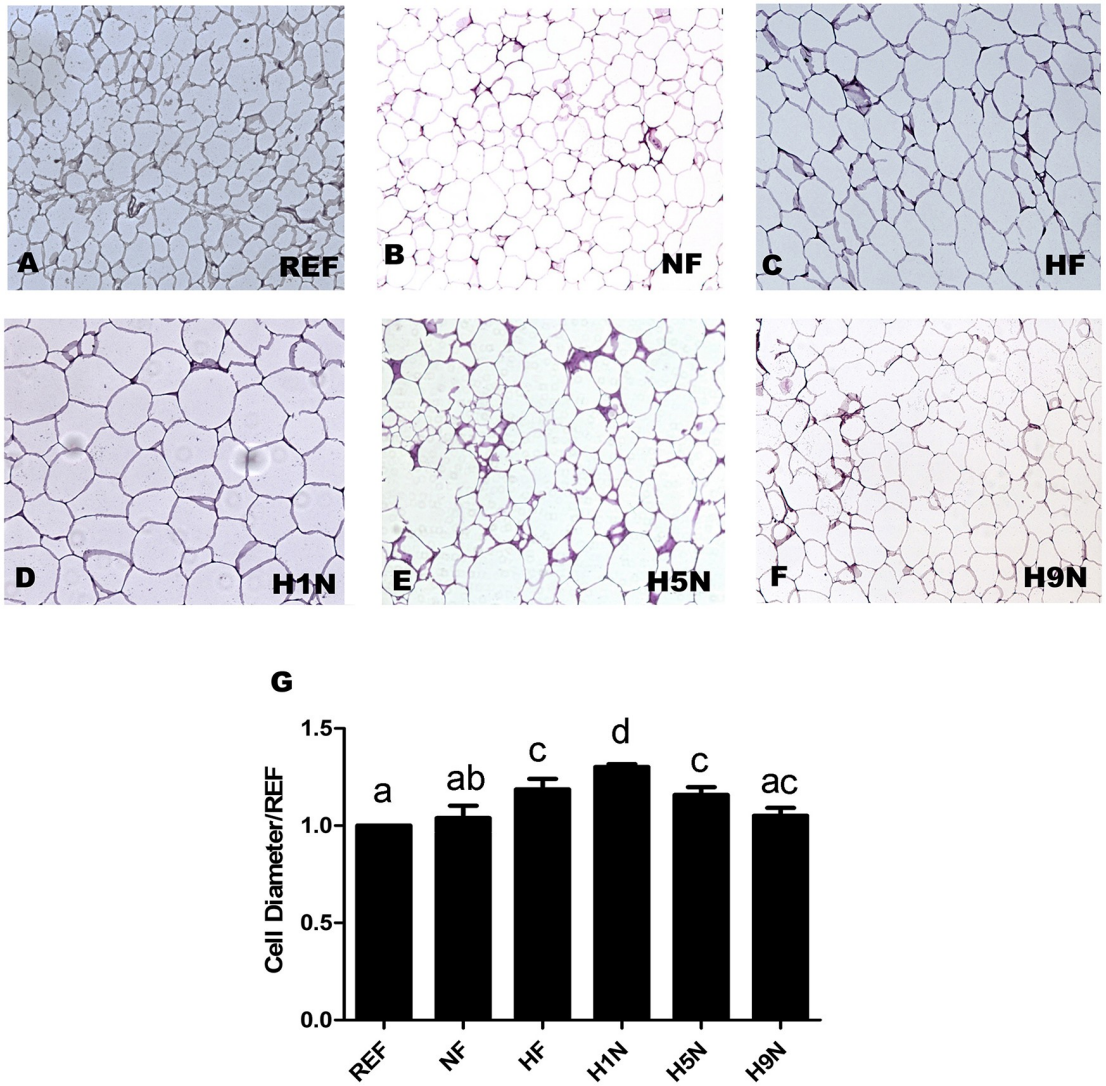
Different maternal diet intervention differentially affected the size of the offspring adipocyte. (A-F) HE staining of visceral adipose tissue. (n = 5–7) (G) Cell diameter measurements of adipocyte normalized against REF group. Data is reported as Mean±SEM, n = 5–7. Different letter indicates significance level of P<0.05 between any two groups.

We next measured the size of adipocytes of each group to determine how different duration of maternal diet intervention effected the enlargement of adipocyte (Fig 2). We did not observe enlarged adipocytes of the NF offspring comparing to the REF mouse, however the maternal HF diet significantly increased the size of adipocytes (HF offspring). Not surprisingly, we observed that the H1N offspring further enlarged the size of their adipocytes comparing to the NF or the HF group, while the H5N adipocyte reduced its diameter to a level similar as the HF adipocyte. Unlike the H5N offspring, the H9N group further reduced adipocyte diameter to a level that was the same as the REF and NF group.

### A long term, but not a short- or medium-term transition recovered overexpression of key modulator genes for fat deposition

**Fig 3:**
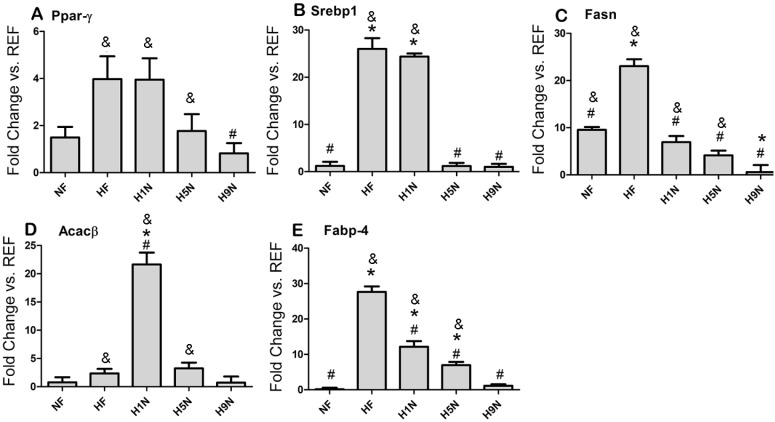
Different maternal diet intervention differentially affected the expression of genes involved in adipogenesis and lipid transportation. (A-E)The adipose tissue expression of *Ppar-γ*, *Srebp1*, *Fasn*, *Acacβ*, and *Fabp4* was measured by real-time PCR. The expression level was normalized against the REF group. Data is reported as Mean±SEM, n = 3–5. The “*” symbol indicates P<0.05 versus the NF group. The “#” symbol indicates P<0.05 versus the HF group. The “&” symbol indicate P<0.05 versus the REF group.

Next, we measured the expression of key modulator genes for adipogenesis and lipid transportation in adipose tissue, aiming to identify the molecular associations between maternal diet intervention and offspring fat deposition (Fig 3). *Ppar-γ* is considered to work as a primary regulator of adipogenesis [38]. *Srebp1c* is highly expressed in adipose tissue and influences adipocyte differentiation by acting upon *Ppar-γ* [39]. We observed significantly enhanced expression of both *Ppar-γ* in the adipose tissue of HF, H1N or H5N offspring comparing to the normal level of REF offspring, although no higher than the NF or HF offspring (Fig 3A). The HF and H1N offspring also had higher expression of *Srebp1* than the NF offspring; however, the *Srebp1* expression in H5N offspring was the same as the NF. Unlike the other treatment groups, the H9N offspring had the same level of *Ppar-γ* and *Srebp1* as the NF group (Fig 3A and 3B).

*Acacβ* and *Fasn* are two genes encoding two rate-limiting enzymes for fatty acid synthesis. The results showed that the post-weaning HF diet remarkably enhanced the adipocyte expression of *Fasn* but not the *Acacβ* in the NF offspring (Fig 3C and 3D, NF group). We also observed significantly higher expression of *Fasn* in HF than the NF offspring (Fig 3C). While the H1N offspring expressed significantly enhanced *Acacβ* in adipose tissue than the REF, NF or the HF offspring (Fig 3D), the H5N offspring had enhanced *Acacβ* level similar as the NF but higher than the REF offspring. In contrast, the H9N offspring had totally recovered level of both genes at a level same as the REF offspring (Fig 3C and 3D).

We further measured the expression of *Fabp4* for fatty acid transportation in adipose. The *Fabp4* level was significantly increased in the HF, the H1N and the H5N groups as compared to the NF group. In contrast, the H9N offspring had completely recovered *Fabp4* expression (Fig 3E). The expression level of all groups ranks from the higher to lower level as HF>H1N>H5N>H9N≈NF.

### A long term, but not a short- or medium-term diet transition recovered macrophage infiltration and reduced presence of crown like structures (CLS) in visceral adipose tissue

**Fig 4:**
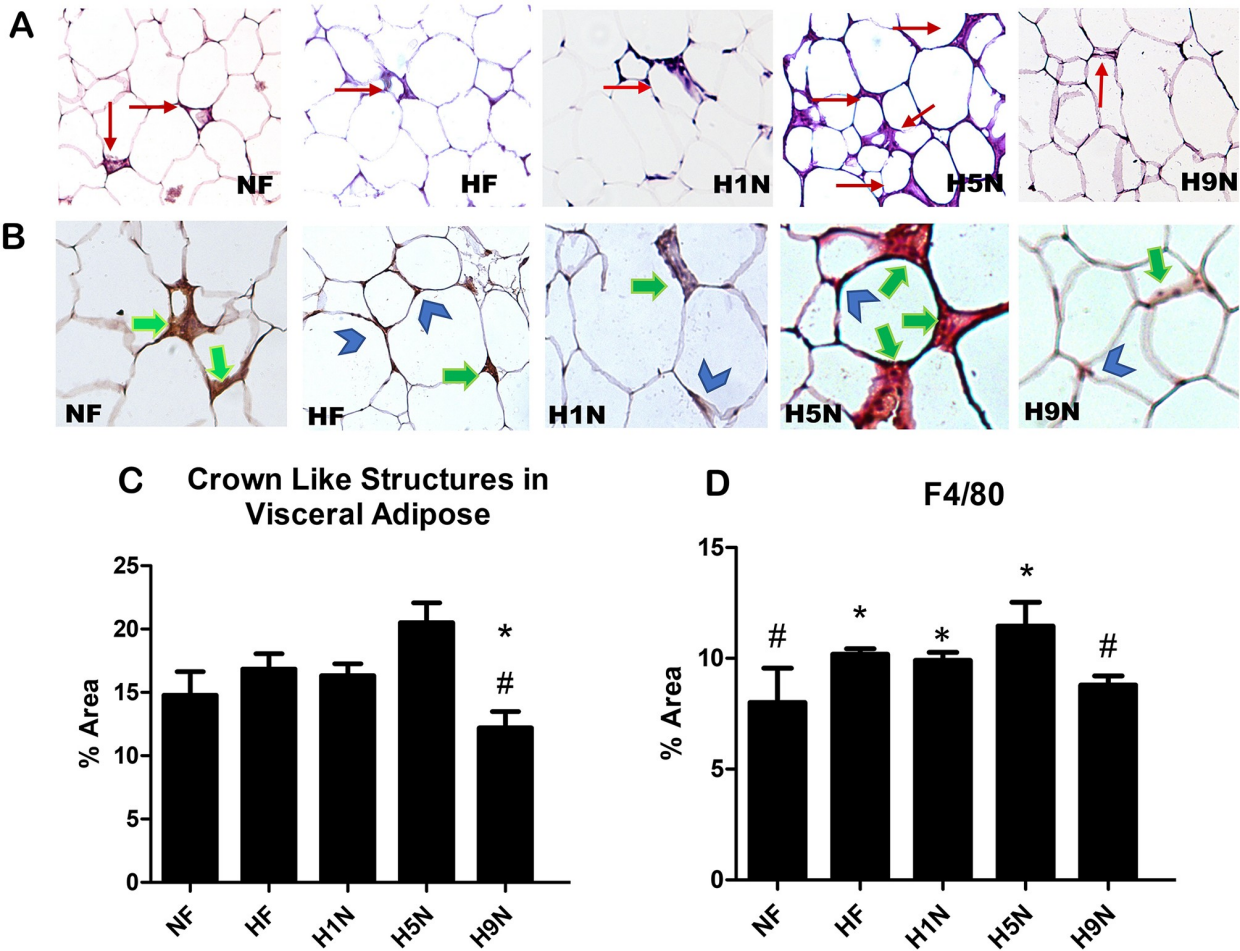
Different maternal diet intervention differentially affected the number of macrophages infiltration. (A) HE staining used to identify CLS (n = 5–7). There was no evident presence of CLS or F4/80 in the REF group. Red arrows indicate CLS. (B) F4/80 staining used to indicate pro-inflammatory macrophages. Green arrows indicate F4/80 present in CLS. Blue arrows indicate F4/80 presence on cell membrane. (C) Absolute percent area of CLS present in visceral adipose tissue determined H9N had a significant decrease compared to both NF and HF groups (n = 5–7, mean ±SEM). (D) Both HF and H5N groups were significantly increased compared to NF group. Data is reported as Mean±SEM, n = 3–5. The “*” symbol indicates P<0.05 versus the NF group. The “#” symbol indicates P<0.05 versus the HF group.

CLS are pockets of lipid spillage resulting from cellular or oxidative damage to adipocyte cell membranes. Consistent with the previous reports [26], the CLS were not visible in the REF offspring. The significantly increased presence of CLS was not observed in any of the three treatment groups, the HF, H1N or H5N groups, comparing to the NF group, although a trend of increasing was noticed. However, The H9N group had a significantly decreased presence of CLS compared to both the HF and NF groups (Fig 4A and 4C).

F4/80 is indicative of activated macrophage presence in adipose tissue [40]. We observed significantly more ATMs of the HF, H1N and H5N offspring than the NF offspring, while the ATM number of the H9N offspring was significantly reduced to an amount similar to the NF offspring (Fig 4B and 4D). Moreover, the expression of F4/80 in the HF, H1N and H5N adipose tissue predominantly localized to the regions of CLS indicating activated pro-inflammatory response of macrophage infiltration. In contrast, the ATMs of the H9N offspring was found both accumulating within the CLS and dissipating around the adipocyte membrane, similar to what occurs in resident macrophage populations of healthy adipose tissue (Fig 4B and 4D).

### A long-term but not a medium- or short-term diet transition reduced the expression of pro-inflammatory cytokines *Tnf-α* and *Il-6* in visceral adipose tissue

**Fig 5:**
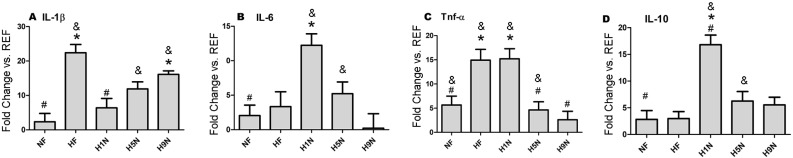
Different maternal diet intervention differentially affected the adipose tissue expression of inflammatory cytokine genes. (A-D)The gene expression level of *Il-1β*, *Il-6*, *Tnf-α* and *Il-10* was measured by real-time PCR. The expression level was normalized against the REF group. Data is reported as Mean±SEM, n = 3–5. The “*” symbol indicates P<0.05 versus the NF group. The “#” symbol indicates P<0.05 versus the HF group. The “&” symbol indicate P<0.05 versus the REF group.

We further assessed inflammatory activity by evaluating the gene expression of inflammatory cytokines involving *Il-6*, *Il-1β*, *Tnf-α* and *Il-10* (Fig 5). We observed enhanced expression of *Tnf-α* but not the other three genes in the NF offspring, suggesting a role of post-weaning HF diet on inducing inflammatory response. Comparing to the NF offspring, the HF offspring had further enhanced *Tnf-α* and *Il-1β* (Fig 5A and 5C). The H1N offspring had lower *Il-1β* expression in its adipose tissue than the HF offspring, which was at the normal level as of the REF group (Fig 5A). In contrast, the H5N offspring had an enhanced *Il-1β* expression higher than the normal but lower than HF offspring, while the *Il-1β* expression of the H9N offspring was the same as the HF offspring. For the *Il-6* level, the adipose tissue had higher level in H1N offspring than in the NF offspring, while it was only higher than the REF level in the H5N offspring. Unlike the H1N and H5N offspring, the H9N adipose tissue expressed a normal level of *Il-6* (Fig 5B). For *Tnf-α*, the HF offspring expressed increased level in adipose tissue than the NF offspring, which was similar as the H1N offspring (Fig 5C). While the H5N adipose tissue expressed a lower than HF but higher than REF level of *Tnf-α*, the H9N adipose tissue expressed normal level of it. Interestingly, we also observed an extremely high level of *Il-10* in the H1N offspring, which was higher than any experimental group. Similar as the H1N, the H5N adipose tissue also enhanced its expression of *Il-10* to level greater than REF but similar to the NF offspring (Fig 5D). Unlike the H1N and H5N offspring, the H9N offspring had normal level of *Il-10*.

### A long-term but not a medium- or a short-term diet transition recovered over-activation of pro-inflammatory signaling pathways

**Fig 6:**
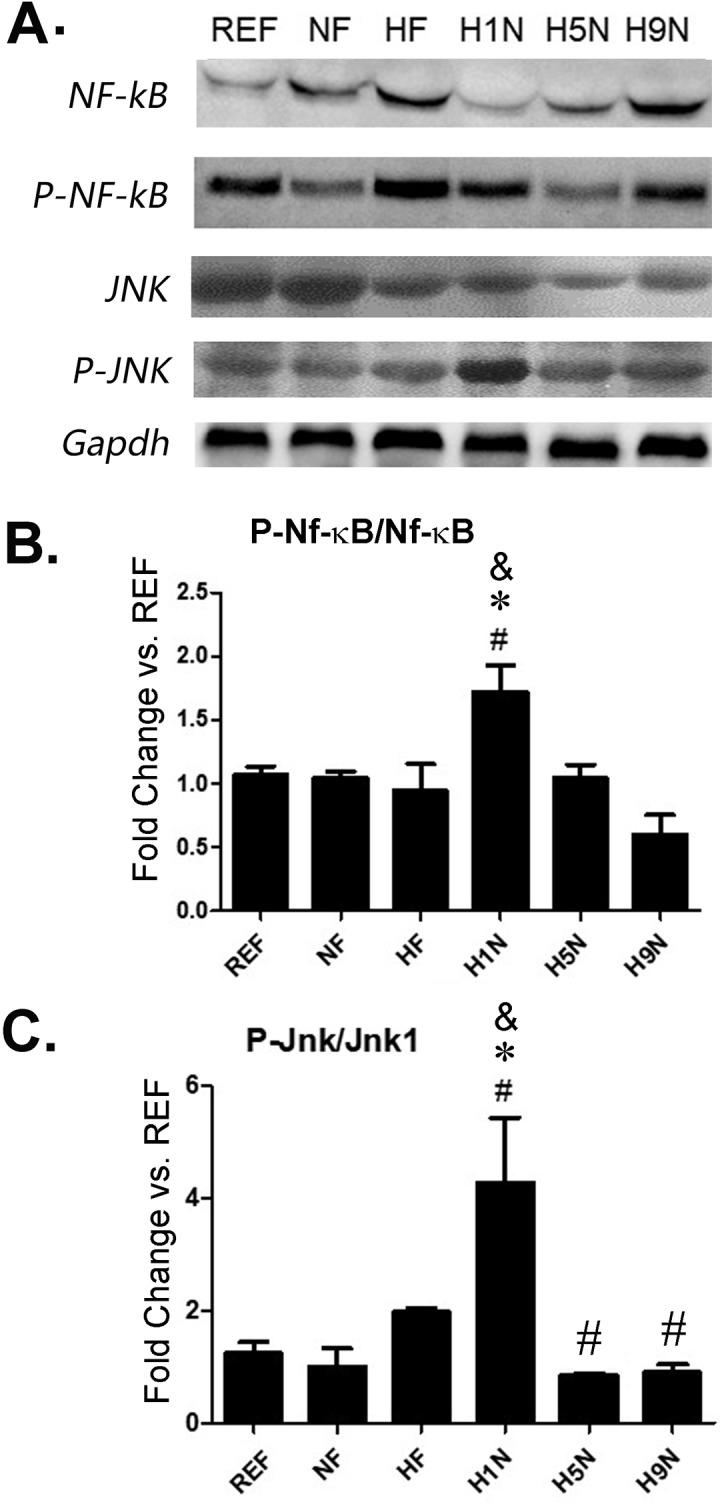
The pro-inflammatory pathways, NF-κB and JNK pathways activation in the offspring adipose tissue was differentially affected by different maternal diet interventions. (A) The expressions of Phospho-NF-κB (65 kDa), NF-κB (65 kDa), JNK (54 kDa) and Phospho-JNK (54 kDa) were detected by Western Blots (n = 3 per group). (B) The Phospho-NF-κB/ NF-κB ratio was calculated and used for statistical analysis. The expression level was normalized against the REF group. Data is reported as Mean±SEM, n = 3–4. The “*” symbol indicates P<0.05 versus the NF group. The “#” symbol indicates P<0.05 versus the HF group. The “&” symbol indicate P<0.05 versus the REF group. (C) The Phospho-JNK/JNK ratio was calculated and used for statistical analysis. Data is reported as Mean±SEM, n = 3–4. The “*” symbol indicates P<0.05 versus the NF group. The “#” symbol indicates P<0.05 versus the HF group. The “&” symbol indicate P<0.05 versus the REF group.

Activation of the JNK and NF-κB signaling pathways are responsible for the increased inflammation [41, 42]. Although we did not observe overactivation of NF- κB signaling indicated by enhanced level of NF-κB and p- NF-κB in both the NF and HF offspring, we did find that the p- NF-κB / NF-κB ratio was significantly higher in H1N group (Fig 6A and 6B).

We did not observe different ratio of pJNK/JNK in the HF offspring between REF, NF or HF offspring (Fig 6A and 6C). The ratio of pJNK/JNK was significantly increased in the H1N group when comparing to the REF, NF or HF offspring. Surprisingly, the JNK activity was depressed in the H5N adipose tissue than compared to the HF offspring (Fig 6A and 6C). The JNK signaling in H9N and H5N adipose tissue was the same as the REF and the NF offspring (Fig 6A and 6C).

## Discussion

Our previous study has indicated that a maternal short-term dietary transition prior to pregnancy (1 week) in female mice significantly exacerbated dysregulated insulin signaling and glucose tolerance induced by post-weaning HF diet in both hepatocytes and adipocytes, more so than treatment with HFD alone [43]. The early onset of symptoms in our mice, independent of maternal glucose intolerance or obesity, prompted the question as to how this maternal diet transition time point may contribute to in-utero metabolic programming, as well as whether longer gaps in transition prior to gestation may play a feasible role in reducing metabolic complications in offspring. Our current study answered that a longer-, but not a medium- or short-term maternal diet intervention resulted in slower body weight gain, maintained glucose tolerance associated with recovered adipocyte hypertrophy and less chronic inflammation in adipose tissue of the male offspring mice challenged with 12-week post-weaning HF diet.

Increased adipocyte size is characteristic of hypertrophy and is an indication of obesity and increased fat mass, which correlates with insulin resistance and aberrant insulin signaling [8, 26, 44, 45]. In our study, both the H1N and H5N offspring but not the H9N offspring displayed hypertrophic adipocytes at different grade and displayed glucose intolerance. Clearly, the adipocyte hypertrophy in H1N and H5N, as well as the HF group correlates with the high expression of adipogenesis genes involving *Ppar-γ*, *Fasn* and *Acaaβ*, suggesting the importance of these genes to predispose offspring adiposity through different maternal diets. Interestingly, the increased amount of visceral adipose tissue was noticed in H9N offspring while adipocyte hypertrophy was not found. This result suggests to us that the adipocyte hyperplasia may be present in the H9N offspring. Hyperplasia is the differentiation of new adipocytes from precursor cells, which primarily occurs in early development [26]. An emerging phenotype of obesity, the “metabolically healthy obese” is thought to have adipose depots with higher rates of hyperplasia and a lower ratio of lipid to cell as compared to traditional models of obesity, indicating that hyper-plasticity of adipose tissue may play a protective role [33, 46–48]. Even though we do not have direct evidence to show that H9N offspring had adipocyte hyperplasia, the normal expression level of adipogenesis gene and the normal size of adipocyte despite of larger amount of visceral adipose tissue highly suggested this possibility. Future study will focus on testing this hypothesis by measuring the activity of IGF-1 signaling and Wnt signaling in adipose tissue of H9N offspring [49–52].

Ours and other’s studies have provided evidence that adipocytes and macrophages interact to promote adipogenesis and pro-inflammatory cytokine-genesis [53–57]. Macrophage infiltration and the presence of CLS are potent indicators of inflammation in adipose tissues [58, 59]. Previous studies have shown that maternal HF-diet predispose offspring obesity and inflammatory changes in the adipose tissue and liver [31, 33]. In our study, significantly more macrophage infiltration, associated with higher adipocyte expression of *Il-1β* and *Tnf-α*, were found in HF group than the NF group suggests that maternal HF-diet predisposes the adipose tissue for inflammation. Interestingly, the H1N and H5N diet did not recover the macrophage infiltration or overexpression of proinflammatory cytokine genes, which were correlated with the abnormal body weight gain, adipocyte hypertrophy and glucose intolerance. However, the H9N offspring had reduced macrophage recruitment and recovered expression of *Il-6*, *Tnf-α*, and *Il-10* associated with retrieved glucose tolerance. These results suggest to us that the maternal diet intervention with a longer-transition period avoids the changes of adipose tissue inflammation and the key pro-inflammatory genes, *Il-6* and *Tnf-α*, are the potential re-programming targets of maternal diet intervention for adipose tissue inflammation.

In our attempt to determine the important molecular signaling pathways perturbed by maternal diet intervention contributing to adipose tissue inflammation, we showed an overactivation of NF-κB and JNK signaling in H1N adipose tissue, but not the other groups. Having observed a high level of chronic inflammation in adipose tissue of the HF and H5N offspring, our results suggested to us that NF-κB and JNK signaling were not the key signaling pathways responsible for adipose tissue inflammation triggered by these two maternal diet interventions. Nonetheless, our data suggested to us that different maternal diet interventions differentially regulate the overexpression of inflammatory cytokines through different molecular signaling. In addition, activated NF-κB and JNK signaling correlates with the more severe adipose tissue inflammation observed in H1N offspring than in the NF offspring, evidenced by more ATMs and higher expression of *Il-1β*, *Il-6*, *Tnf-α* and *Il-10*. Previous studies have demonstrated an essential role of the Toll-like receptor-4 signaling pathway in triggering and promoting metabolic inflammation [23, 60–64]; hence, this will be a focus of our future study.

Our study provides evidence that a long-term maternal diet intervention can avoid the offspring adipose tissue inflammation. A relationship between adipose tissue macrophage accumulation and adipocyte size has been demonstrated in many adipose tissue depots [59]. The recruitment of adipose tissue macrophages is triggered when fat cells reach a critical size [59, 65, 66]. According to this theory, the effect of H9N to avoid adipose tissue inflammation might be due to the smaller size of adipocyte. Consistently, the H9N offspring had lower expression of genes involved in adipogenesis associated with less amount of adipose tissue macrophages. On the other hand, the HF, H5N and H1N offspring all enlarged the adipocyte size associated with increased expression of adipogenesis genes and more macrophage infiltration. Thus, re-programing the adipocyte by maternal diet intervention seems to be an earlier step than re-programming the adipose tissue inflammation. However, re-programming the adipose tissue inflammation is obviously not dependent on the size of the adipocytes, evidenced by the fact that the H5N had the most macrophage infiltration, however only moderately increased the gene expression of the adipogenesis and pro-inflammatory cytokines. Recent elegant studies have linked epigenetic regulation, especially the DNA methylation, with adipose tissue inflammation [67–69]. Thus, identifying key genes in adipose tissue inflammation, responsible for different maternal diet interventions, will be one of the focus of our future study.

Metabolic memory, is theorized as the memory the body retains of a metabolic insult such as prolonged changes in microcirculation due to hyperglycemia in diabetes, even after recovery such as when blood glucose becomes better controlled, [70]. This theory has been researched for its importance to promote more aggressive and earlier treatment for deterring advancement of metabolic diseases [70]. In our study, we witnessed the recovery of a distinct metabolic phenotype, which effectively reversed the damaging effects of maternal HFD administration via a maternal diet intervention with a long-term transition period before gestation. A stepwise reduction in phenotype between the H1N, H5N, and H9N groups would be observed if maternal metabolic memory was time-dependent. Yet the H5N group displayed exacerbated glucose intolerance and increased macrophage infiltration compared to all other groups. Literature has noted that HFD intake immediately prior to conception has longstanding influences on activation of maternal lipogenesis genes in the placenta, leptin generation and circulation, and adiposity affecting offspring long term, that may provide a more concrete characterization of how alterations in maternal diet influence reprogramming *in utero* [71–73].

Overall, our study was first to evaluate how maternal diet transition at different time points prior to pregnancy effects offspring outcomes of inflammation and obesity in the visceral adipose tissue. Visceral adipose tissue is a driver of metabolic complications such as Type 2 diabetes mellitus, non-alcoholic fatty liver disease, and metabolic syndrome. Understanding how maternal dietary intake affects offspring risk, via either exacerbating or recovering inflammatory phenotype, has potent implications for reducing intergenerational risk of disease. Our work demonstrates the ability of properly timed maternal diet intervention to reduce risk yet effectively illustrates that maternal diet, if poorly timed, can exacerbate negative offspring phenotypes, in some instances more so than without intervention.
